# Supplementary material for: Large-scale whole exome sequencing studies identify two genes,CTSL and APOE, associated with lung cancer
Source: PLoS Genet. 2023 Sep 22;19(9):e1010902. doi: 10.1371/journal.pgen.1010902 (PMC10516417; doi:10.1371/journal.pgen.1010902)
Supplement: S1 Text — (DOCX) [file pgen.1010902.s001.docx]

**S1 Text. Method Supplement**

1. **Derivation of Bayes Factor (BF) Test**

In brief, for each gene *G*, the BF test statistic is defined as a ratio of marginal likelihoods of the alternative hypothesis (m_1_) that gene *G* is associated with the disease (i.e., lung cancer case-control status) over the null hypothesis (m_0_) that gene *G* is not associated,

$BF=\frac{m_{1}\left( X \right)}{m_{0}\left( X \right)}=\frac{\prod_{i=1}^{2} \int_{P_{i}} f\left( X_{i} | P_{i} \right)\int_{\theta_{i}} g\left( P_{i} | \theta_{i} \right)\pi\left( \theta_{i} \right)d\theta_{i}dP_{i}}{\int_{P} f\left( X | P \right)\int_{\theta} g\left( P | \theta\right)\pi\left( \theta\right)d\theta dP} \left( 1 \right)$,

where $X\equiv(X_{1},X_{2})\equiv(x_{1},\ldots, x_{n})$denotes the RV counts among $n$ individuals, and $X_{1}$and $X_{2}$ the RV counts in cases and controls, respectively; $P\equiv(P_{1},P_{2})\equiv(p_{1},\ldots, p_{n})$ denotes the RV proportions within the gene over the $n$ individuals, where $P_{1}$and $P_{2}$ are the proportions in cases and controls, respectively. We assume that for an individual $k$, $x_{k}\sim Binomial(m,p_{k})$, where $m$ is the total number of RV sites within the gene *G*, assuming $m\geq20$. Further, *f(.)* denotes the binomial distribution function, *g(.)* the prior density function for $P$, and $\pi(.)$ the prior density function which depends on the hyper-parameters $\theta.$ Our goal is to assess whether there is a difference in RV counts between cases and controls for gene *G* by comparing the null hypothesis (*H_0_*) of no gene association to the alternative of a gene association (*H_1_*) as

$$H_{0}:\theta_{1}=\theta_{2}=\theta vs. H_{1}:\theta_{1}\neq\theta_{2},$$

using the BF statistic [1]. The BF is larger when there is a large difference in RV counts between cases and controls.

1. **BF test with informative prior**

An original feature of the BF is the possibility to introduce an “informative” prior to gain power to detect gene-based associations. This “informative” BF for a gene G is formulated as

$${BF}_{inf}=2\log(BF)-2\log\left( P_{inf} \right),$$

where *P_inf_* captures allelic differences between cases and controls for gene G.

We initially proposed to derive *P_inf_* from a Kolmogorov-Smirnov (KS) test, leading to the *BF_KS_* statistic. We have demonstrated in the Theorem 1 from [1] that incorporating the KS test P-value as an informative prior, under the null hypothesis (*H_0_*), ${2logBF}_{inf}$follows a distribution of $\chi^{2}(3) [1].$The idea is to conduct a series of single RV tests for all RVs within a gene G and then to compare the resulting distribution of *P* values across all RVs to a null distribution by a one-sided one-sample KS test. The null distribution is empirically estimated using all the genes across the genome. For the ILCCO study, which involves a balanced case-control design, the single RV test is based on comparing the mean RV count in cases vs. controls and assuming a Poisson distribution for this RV count at each RV site [1]. For UK Biobank, which involves a very unbalanced design (very small fraction is cases) and with a high proportion of RV counts equal to 0 in cases, the single RV association was based on Fisher’s exact test. For those RVs with extremely low MAF, the *P* values from Fisher’s exact test were equal to 1 and were excluded from the KS test.

As an alternative to the *BF_KS_* statistic, we also propose here to derive *P_inf_* from a SKAT test statistic [2] as a secondary analysis, leading to the *BF_SKAT_* statistic. SKAT is a score-based variance component test, which uses the mixed effects model framework wherein a phenotype is regressed on a set of variants from one gene or region. Here, only RVs are considered. It has been shown that SKAT is powerful to detect RVs in situations where these RVs have different directions and magnitudes of effects. As the SKAT statistic can be thought as of a weighted sum of individual variant score statistics, it is also sensitive to detect gene-based association when a small proportion of causal variants explain the phenotype variability. To account for the unbalanced case-control ratio in UK Biobank data, we applied a robust version of SKAT for gene-based analysis, wherein the single variant score test statistic is calibrated based on saddle point approximation and efficient resampling [3]. We note that although only European-descent individuals were included in the discovery and replication studies, a within-Europe population structure could still influence the results of association tests, especially for methods allowing variants to have opposite directions of effect [4]. To avoid this potential confounding effect, the SKAT test was adjusted after including the top 5 PCs in the regression model. Besides, analyses in UK Biobank were also adjusted for sex and age.

*BF_KS_* and *BF_SKAT_* statistics might therefore prove useful for both gene-based and single RV-based association studies. Indeed, the BF with noninformative prior compares the overall RV count between cases and controls, whereas the KS component in *BF_KS_* is based on allelic distribution differences between cases and controls at individual sites within the region, and the SKAT component in *BF_SKAT_* is based on the contribution of each RV site to the phenotype variability.

We have previously shown that the BF statistic (with noninformative prior) and *P_inf_* derived from the KS test were uncorrelated [1]. We further show that this is also the case for the BF statistic (with noninformative prior) and *P_inf_* derived from the SKAT score-test (randomly selected 100 gene sets, each including 1000 genes, the mean of the Kendall correlation coefficient is 0.11). These properties lead 2log(*BF_KS_*) and 2log(*BF_SKAT_*) each to follow a $\chi^{2}$ distribution with 3 degrees of freedom as the number of cases and the number of controls go to infinity. Therefore, a *P* value can be derived directly from our BF approach without the need to use re-sampling techniques.

1. **Sensitivity analysis: BF tests adjusted for covariates**

To adjust for potential confounding variables, e.g., related to population structure, we extended the BF test (with noninformative prior) by incorporating a beta regression model of hyper-parameter $\theta_{i}$ that allows to account for confounding variables. As illustrated in [1], for group i, $i\in\left\{ 1,2 \right\}$ (1 for the control group, 2 for the case group) and individual k, $k\in\left\{ 1,...,N_{i} \right\}$, we assume that rare variant counts $x_{ik}$

$x_{ik}\sim Binomial(m,p_{ik})$ and $p_{ik}|\theta_{i}\sim beta(\eta_{ik},K)$,

where $m$ is the total number of RV sites within the gene *G*, assuming $m\geq20$. Here the beta distribution is parametrized in terms of mean (denoted by $\eta_{ik}$) and precision (denoted by $K$) parameter. The following beta regression is considered for $\eta_{ik}$,

$g\left( \eta_{ik} \right)=\alpha_{0}+\alpha_{1}\mathbb{l}_{\{i=2\}}+Z_{ik}^{T}\alpha_{2}$,

where $g(.)$ is the logit function, $Z_{ik}^{T}$ represents covariates matrix, including sex, age, smoking, top 5 PCs. We further define $g\left( \eta_{1} \right)=\alpha_{0}$for controls and $g\left( \eta_{2} \right)=\alpha_{0}+\alpha_{1}$ for cases and assume a hyper-prior for $\eta_{i}\sim beta(\eta_{i}^{*},K_{i}^{*})$. Our goal is to use BF to test

$$H_{0}:\eta_{1}=\eta_{2}=\eta vs. H_{1}:\eta_{1}\neq\eta_{2}.$$

The marginal likelihood of the data X under $H_{0}$ is

$$m_{0}\left( X | K,\alpha_{2}, \eta^{*},K^{*} \right)=\int_{\eta} \prod_{i=1}^{2} \prod_{k}^{N_{i}} [\binom{n_{ik}}{x_{ik}}\frac{B\left( x_{ik}+K\eta_{ik},n_{ik}-x_{ik}+K\left( 1-\eta_{ik} \right) \right)}{B\left( K\eta_{ik},K\left( 1-\eta_{ik} \right) \right)}]|_{\eta_{ik}=\frac{exp\left( logit\left( \eta\right)+Z_{ik}^{T}\alpha_{2} \right)}{1+exp\left( logit\left( \eta\right)+Z_{ik}^{T}\alpha_{2} \right)}}$$

$\frac{\eta^{K^{*}\eta^{*}-1}{(1-\eta)}^{K^{*}(1-\eta^{*})-1}}{B(K^{*}\eta^{*},K^{*}(1-\eta^{*}))}d\eta$.

The marginal likelihood of the data X under $H_{1}$ is

$$m_{1}\left( X | K,\alpha_{2}, \eta_{1}^{*},K_{1}^{*},\eta_{2}^{*},K_{2}^{*} \right)=\prod_{i=1}^{2} \int_{\eta_{i}} \prod_{k}^{N_{i}} [\binom{n_{ik}}{x_{ik}}\frac{B\left( x_{ik}+K\eta_{ik},n_{ik}-x_{ik}+K\left( 1-\eta_{ik} \right) \right)}{B\left( K\eta_{ik},K\left( 1-\eta_{ik} \right) \right)}]|_{\eta_{ik}=\frac{exp\left( logit\left( \eta_{i} \right)+Z_{ik}^{T}\alpha_{2} \right)}{1+exp\left( logit\left( \eta_{i} \right)+Z_{ik}^{T}\alpha_{2} \right)}}$$

$\frac{{\eta_{i}}^{K_{i}^{*}\eta_{i}^{*}-1}{(1-\eta_{i})}^{K_{i}^{*}(1-\eta_{i}^{*})-1}}{B(K_{i}^{*}\eta_{i}^{*},K_{i}^{*}(1-\eta_{i}^{*}))}d\eta_{i}$.

The BF is the ratio of marginal likelihood under $H_{1}$and under $H_{0}$

$BF=\frac{m_{1}\left( X | K,\alpha_{2}, \eta_{1}^{*},K_{1}^{*},\eta_{2}^{*},K_{2}^{*} \right)}{m_{0}\left( X | K,\alpha_{2}, \eta^{*},K^{*} \right)}$.

In this expression, we consider a fixed $K$ and $\alpha_{2}$ and estimate their MLE $\tilde{K}$ and $\tilde{\alpha}_{2}$from the whole sample under $H_{0}$. Therefore,

$BF=\frac{m_{1}\left( X | \tilde{K},\tilde{\alpha}_{2}, \eta_{1}^{*},K_{1}^{*},\eta_{2}^{*},K_{2}^{*} \right)}{m_{0}\left( X | \tilde{K},\tilde{\alpha}_{2}, \eta^{*},K^{*} \right)}$.

Following the same principle in the Theorem 1 in [1], the integral part in the BF formula is calculated using Laplace approximation, and BF test statistic adjusted for covariates similarly follows $\chi^{2}(1)$ distribution under $H_{0}$.

The BF with informative KS prior also adjusts the KS component for covariates in the single RV association test. The single RV score test statistic is calibrated based on saddle point approximation and efficient resampling [3] and is adjusted for age, sex, smoking, and top 5 PCs. When SKAT region-based test is used as prior in the BF test, we adjusted for the same covariates using either the original SKAT method [4] for the ILCCO study or the robust SKAT approach [3] for the UK Biobank data.

We applied the adjusted *BF_KS_* (both BF and KS prior are adjusted for covariates) and *BF_SKAT_* (both BF and SKAT prior are adjusted for covariates) on the CTSL and APOE genes to assess the sensitivity of the association tests on the covariate adjustment.

**References**

1. Xu J, Xu W, Briollais L. A Bayes factor approach with informative prior for rare genetic variant analysis from next generation sequencing data. Biometrics. 2021 Mar;77(1):316-328.
2. Lee S, Wu MC, Lin X. Optimal tests for rare variant effects in sequencing association studies. Biostatistics. 2012;13(4):762-775. doi:10.1093/biostatistics/kxs014
3. Zhao Z, Bi W, Zhou W, VandeHaar P, Fritsche LG, Lee S. UK Biobank Whole-Exome Sequence Binary Phenome Analysis with Robust Region-Based Rare-Variant Test. Am J Hum Genet. 2020 Jan 2;106(1):3-12. doi: 10.1016/j.ajhg.2019.11.012. Epub 2019 Dec 19. PMID: 31866045; PMCID: PMC7042481.
4. Wu MC, Lee S, Cai T, Li Y, Boehnke M, Lin X. Rare-variant association testing for sequencing data with the sequence kernel association test. Am J Hum Genet. 2011 Jul 15;89(1):82-93. doi: 10.1016/j.ajhg.2011.05.029. Epub 2011 Jul 7. PMID: 21737059; PMCID: PMC3135811.
5. Zawistowski M, Reppell M, Wegmann D, St Jean PL, Ehm MG, Nelson MR, Novembre J, Zöllner S. Analysis of rare variant population structure in Europeans explains differential stratification of gene-based tests. Eur J Hum Genet. 2014 Sep;22(9):1137-44. doi: 10.1038/ejhg.2013.297. Epub 2014 Jan 8. PMID: 24398795; PMCID: PMC4135410.
