# Supplementary material for: Large-scale whole exome sequencing studies identify two genes,CTSL and APOE, associated with lung cancer
Source: PLoS Genet. 2023 Sep 22;19(9):e1010902. doi: 10.1371/journal.pgen.1010902 (PMC10516417; doi:10.1371/journal.pgen.1010902)
Supplement: S1 Table — (DOCX) [file pgen.1010902.s002.docx]

S1 Table. Results of gene-based analysis using adjusted *BF_KS_* test in the discovery and replication

|  | Discovery (ILCCO) | | | Replication (UK Biobank) | | |
| --- | --- | --- | --- | --- | --- | --- |
|  | BF^*#^ p | KS^*^ p | *BF_KS_*^*^ p | BF^*#^ p | KS^*^ p | *BF_KS_*^*^ p |
| CTSL | 3.68E-03 | 4.77E-04 | 2.84E-05 | 6.30E-04 | 4.28E-01 | 3.88E-03 |

*Adjusted for age, sex, smoking (never smokers vs. others), and top 5 PCs

^#^BF with noninformative prior
