# Supplementary material for: Large-scale whole exome sequencing studies identify two genes,CTSL and APOE, associated with lung cancer
Source: PLoS Genet. 2023 Sep 22;19(9):e1010902. doi: 10.1371/journal.pgen.1010902 (PMC10516417; doi:10.1371/journal.pgen.1010902)

**S1 Figure.** Population Structure shown in top 3 principal components (PCs), PC1, PC2, and PC3 of each cohort in ILCCO study, PC1 vs. PC2 (I), PC1 vs. PC3 (II), and PC2 vs. PC3 (III). The graphs A, B, C, D represent Toronto, HSPH-MGH, Liverpool and IARC sites involved in ILCCO study, respectively. The patterns shown in graph A and B are similar, suggesting the ethnicity backgrounds of Toronto and HSPH-MGH samples are very similar. Liverpool cohort has much more homogeneous ancestry background than the other cohorts.

I.


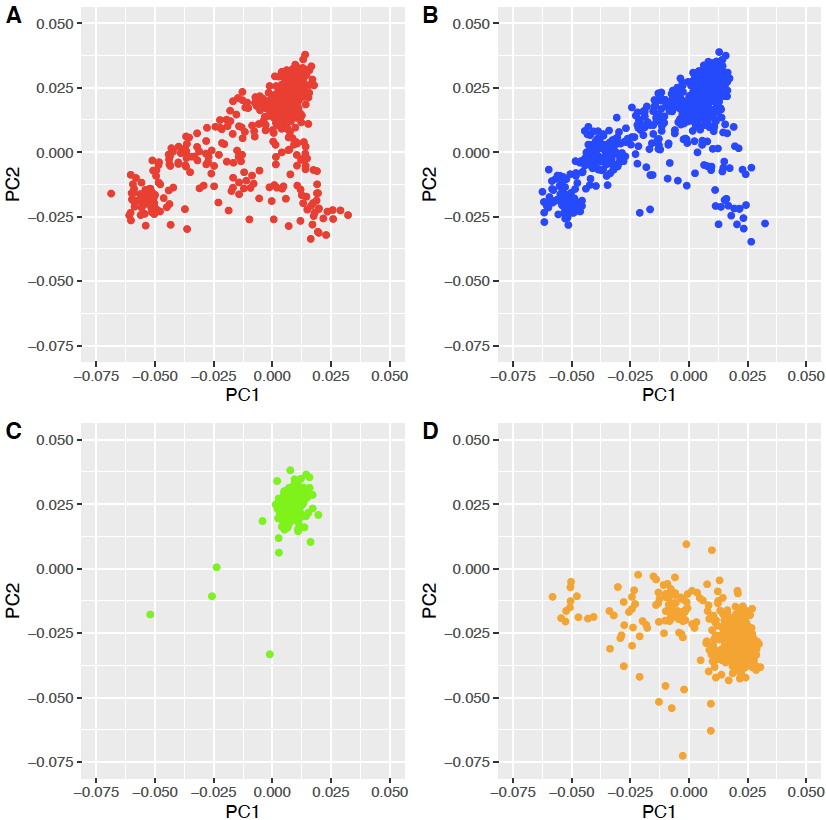


II.


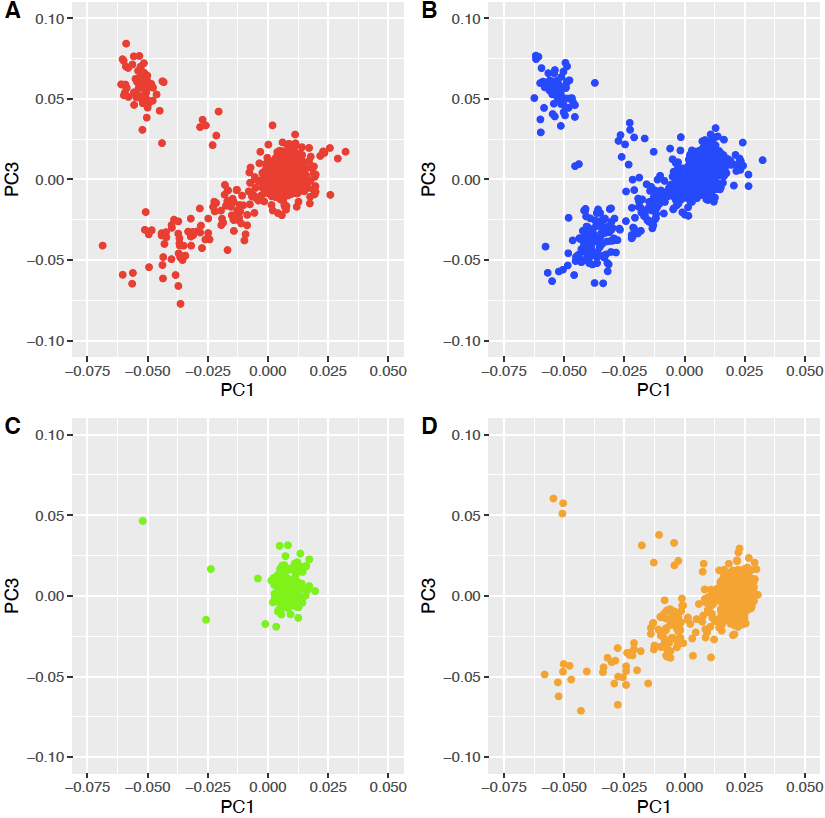


III.


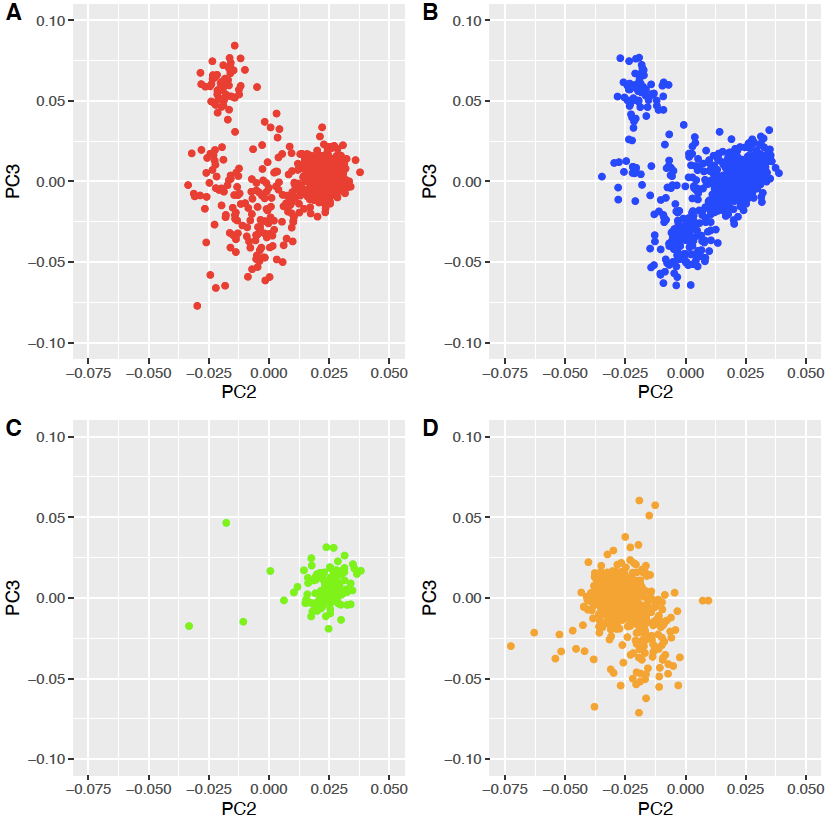

Supplement: S1 Fig — (DOCX) [file pgen.1010902.s004.docx]
