# Supplementary material for: Large-scale whole exome sequencing studies identify two genes,CTSL and APOE, associated with lung cancer
Source: PLoS Genet. 2023 Sep 22;19(9):e1010902. doi: 10.1371/journal.pgen.1010902 (PMC10516417; doi:10.1371/journal.pgen.1010902)

**S2 Figure.** Relationship between QUAL and mean GQ vs. Ts/Tv ratio. Ts/Tv ratio is usually an indicator of overall SNV quality. It is known that Ts/Tv ratio is around 2 across the entire genome, and around 3 in the exome region. Based on figure I and II, we filtered variants with QUAL<100 or mean GQ<50.

I.


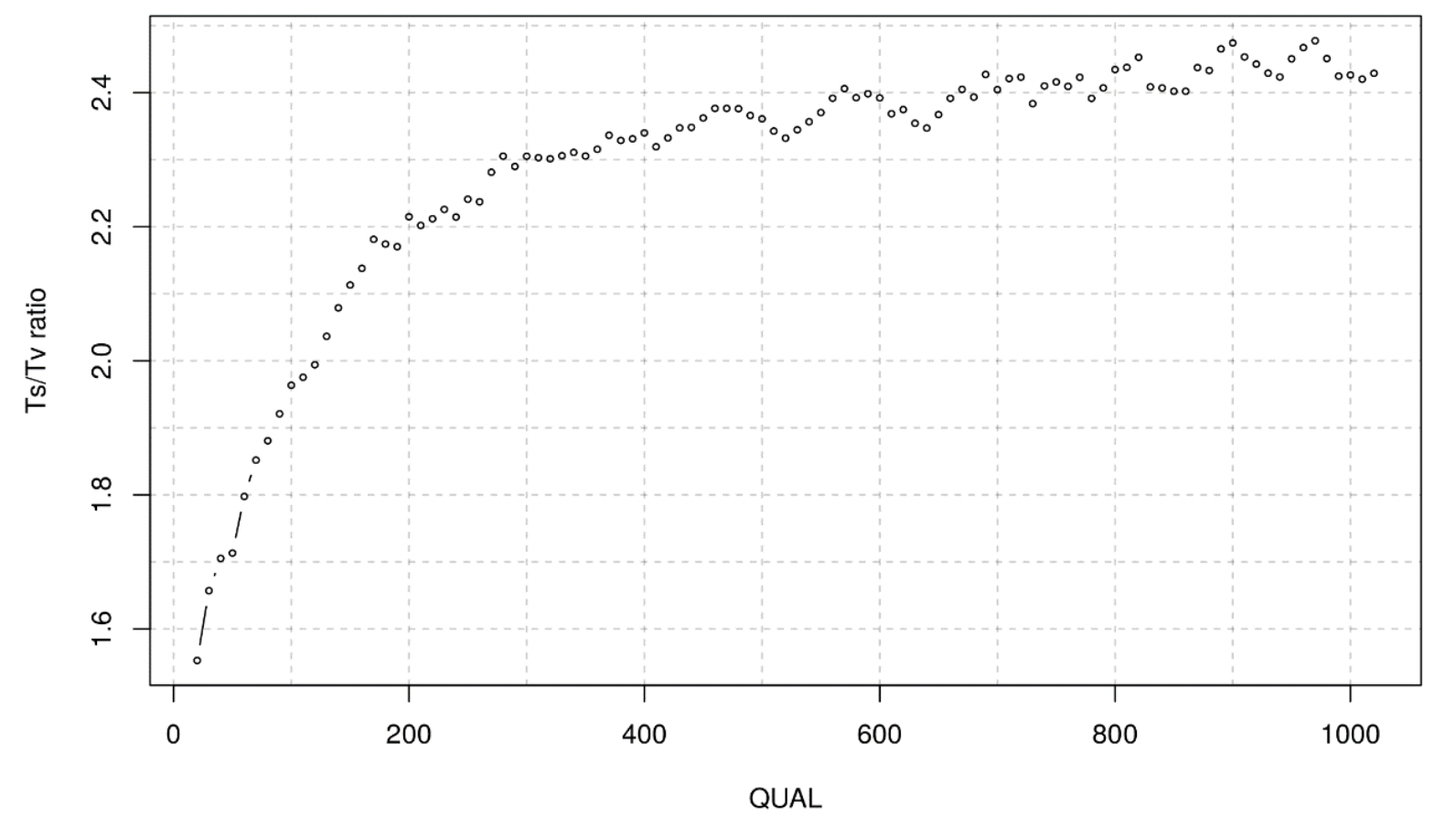


II.


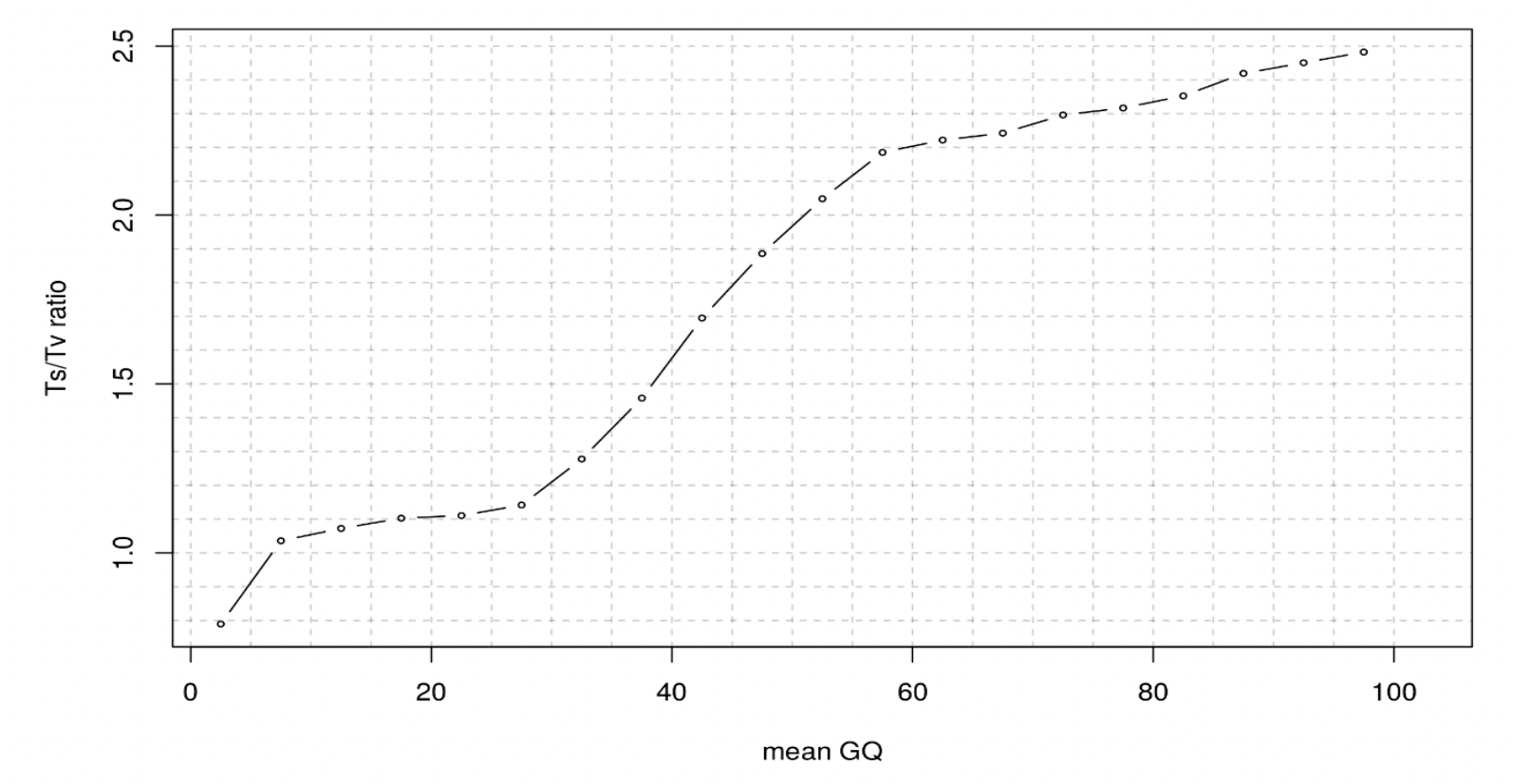

Supplement: S2 Fig — (DOCX) [file pgen.1010902.s005.docx]
