# Supplementary material for: Large-scale whole exome sequencing studies identify two genes,CTSL and APOE, associated with lung cancer
Source: PLoS Genet. 2023 Sep 22;19(9):e1010902. doi: 10.1371/journal.pgen.1010902 (PMC10516417; doi:10.1371/journal.pgen.1010902)
Supplement: S3 Fig — (DOCX) [file pgen.1010902.s006.docx]

**S3 Figure.** Genetic region of rs112682750 (pos: 87727608, build 38) within *CTSL* gene.


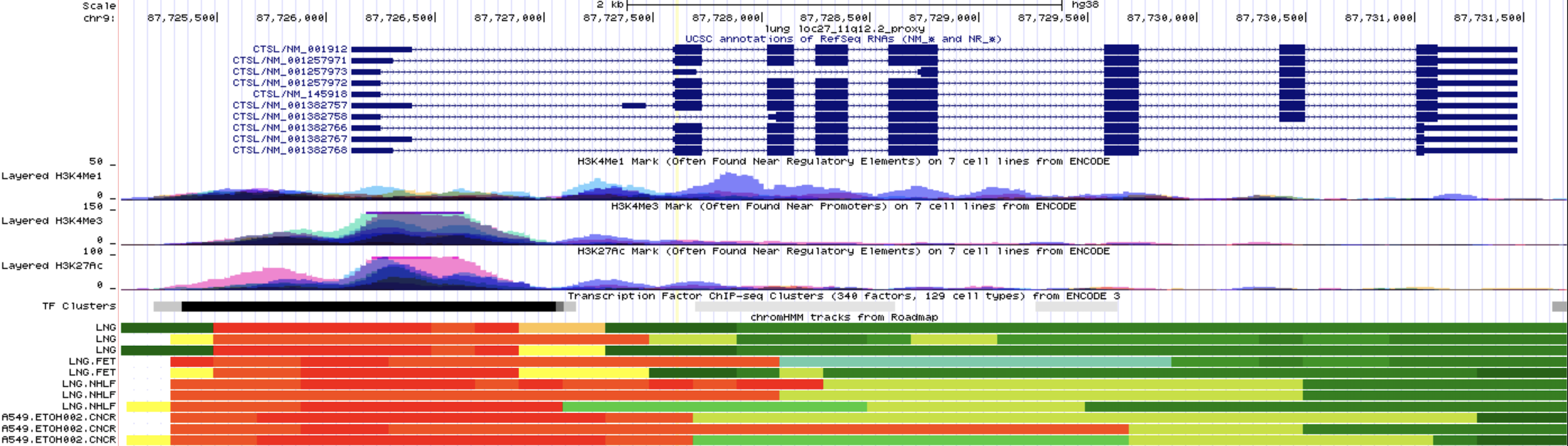
 The colors of the horizontal bars represent the chromatin state annotation of lung-related tissues[1] (LNG, lung; LNG.FET, fetal lung; LNG.NHLF, lung fibroblast; A549.ETOH002.CNCR, lung carcinoma), where “(orange) red bar” denotes TSS-proximal promoter state and “(dark/lime) green” denotes transcribed state.

**Reference**

1. Roadmap Epigenomics Consortium, Kundaje A, Meuleman W, Ernst J, Bilenky M, Yen A, Heravi-Moussavi A, Kheradpour P, Zhang Z, Wang J, Ziller MJ, Amin V, Whitaker JW, Schultz MD, Ward LD, Sarkar A, Quon G, Sandstrom RS, Eaton ML, Wu YC, Pfenning AR, Wang X, Claussnitzer M, Liu Y, Coarfa C, Harris RA, Shoresh N, Epstein CB, Gjoneska E, Leung D, Xie W, Hawkins RD, Lister R, Hong C, Gascard P, Mungall AJ, Moore R, Chuah E, Tam A, Canfield TK, Hansen RS, Kaul R, Sabo PJ, Bansal MS, Carles A, Dixon JR, Farh KH, Feizi S, Karlic R, Kim AR, Kulkarni A, Li D, Lowdon R, Elliott G, Mercer TR, Neph SJ, Onuchic V, Polak P, Rajagopal N, Ray P, Sallari RC, Siebenthall KT, Sinnott-Armstrong NA, Stevens M, Thurman RE, Wu J, Zhang B, Zhou X, Beaudet AE, Boyer LA, De Jager PL, Farnham PJ, Fisher SJ, Haussler D, Jones SJ, Li W, Marra MA, McManus MT, Sunyaev S, Thomson JA, Tlsty TD, Tsai LH, Wang W, Waterland RA, Zhang MQ, Chadwick LH, Bernstein BE, Costello JF, Ecker JR, Hirst M, Meissner A, Milosavljevic A, Ren B, Stamatoyannopoulos JA, Wang T, Kellis M. Integrative analysis of 111 reference human epigenomes. Nature. 2015 Feb 19;518(7539):317-30. doi: 10.1038/nature14248. PMID: 25693563; PMCID: PMC4530010.
