# Supplementary material for: Large-scale whole exome sequencing studies identify two genes,CTSL and APOE, associated with lung cancer
Source: PLoS Genet. 2023 Sep 22;19(9):e1010902. doi: 10.1371/journal.pgen.1010902 (PMC10516417; doi:10.1371/journal.pgen.1010902)
Supplement: Attachment — Submitted filename: Reply.docx [file pgen.1010902.s007.docx]

We would like to thank the Editorial Office and reviewers for their very valuable comments and suggestions. We have revised our manuscript taking these into considerations, as detailed below. The page and line number indicated in this document follow the numbering system used in the clean version of the manuscript (no track changes).

**Editor**

**From my editorial perspective, I read the Appendix section detailing the maths behind the test but I could not manage to get an intuitive understanding of the differences between the BF test and the more traditional options, for example the commonly used SKAT test. Additional background in the introduction or the methods sections to explain in easy to understand terms (a toy example exemplifying a situation where the tests diverge perhaps?) what the differences are (and based on Tables 3 and 4 it is substantial) would be something I would like to see in a revised version.**

Compared to the commonly-used SKAT gene-based test [15], our BF approach is more sensitive to an excess of small p-values from single RV tests within each gene while SKAT has better power to detect genes exhibiting systematic allelic differences between cases and controls across all RVs. This difference was discussed in details in Xu, et al. [11] and illustrated on two genes that showed large discrepancy in overall ranking when applying either approach [11]. The description of the difference between BF and SKAT is added in the methods section of the MS (Page 9, line 148).

**I also like the idea of reviewer 1 of a permutation analysis to understand the level of noise in the analyses (which would in turn justify the choice of thresholds). Given that the results are not extremely significant, it seems to me that it is important to be as transparent as possible about the expected distribution of the test statistic.**

We took the reviewer’s suggestion and have now performed permutation analysis to determine the threshold in the discovery and validation studies for the BF with KS prior (i.e., our main analysis). This is included in the MS (page 10, line 173).

**Please be aware that our** [**data availability policy**](http://journals.plos.org/plosgenetics/s/data-availability) **requires that all numerical data underlying graphs or summary statistics are included with the submission, and you will need to provide this upon resubmission if not already present. In addition, we do not permit the inclusion of phrases such as "data not shown" or "unpublished results" in manuscripts. All points should be backed up by data provided with the submission.**

**Comments to the Authors:
Please note here if the review is uploaded as an attachment.**

**Reviewer #1: Xu et al perform a gene-based analysis of whole exome sequence data for lung cancer. They first analyse data from the International Lung Cancer Consortium (1000 cases, 900 controls), then from UK Biobank (600 cases, 170k controls). They claim to identify two genes significantly associated with lung cancer risk, which combined contain 6 associated rare variants.

This is an interesting study, and it appears that the ILCC WES data are the largest WES data available for lung cancer (I could not find larger based on a quick Google). I note that the original analysis of these data (Ref 10) had limited success (only 5 putatively significant single-SNP associations, and no significant genes), so it would be good if a reanalysis using a more powerful tool produced significant findings.

In general, I feel the gene-based results are valid (i.e., that two genes achieve global significance). I disagree that within these genes, 6 individual SNPs are significant (because I disagree with using the FDR to determine significance). However, this is not very concerning, because the single-SNP analysis is only a secondary analysis.**

We agree that further validation of the individual variants is needed. We mainly used FDR as a less conservative approach to highlight the important single variants within the genes discovered compared to the more commonly-used Bonferroni approach.

**Note that the authors often talk about controlling the false discovery rate (FDR), which initially made me skeptical (because FDR-based significance thresholds can be too relaxed). However, I believe that in practice, their "global FDR" threshold is equivalent to controlling the family-wide error rate (FWER), which is standard. If I am correct, I suggest referring to the threshold as a FWER to avoid confusion.**

We agree that our approach was meant to control the FWER instead of FDR. To determine the threshold of p-values in the discovery and validation sets, we now used a permutation approach, as suggested by the reviewer. Compared to our previous approach, the permutation analysis is more robust to the deviation from the distribution of BF on the real data under the null hypothesis but led to more conservative thresholds (Page 10, line 173). **Major comments

1 - As I say above, I was initially concerned about the significance thresholds used for the gene-based test. However, it seems to me that setting alpha=.05/13872 in Equation on Page 9 corresponds to ensuring the FWER from the meta analysis (i.e., combining gene-based p-values from discovery and replication data) is not higher than 0.05. In fact, your thresholds may be slightly too strict, because your replication analysis considers only a subset of genes (although I do not suggest relaxing to compensate!). Similarly, at first read, I thought the choices of per-study thresholds (i.e., 6e-4 and 5e-2) were suspicious. For example, why pick 6e-4 as the discovery threshold, and not 6e-5? However, I believe changing the threshold will not lead to false positives (because utlimately they do not change the meta analysis threshold), and can instead only lead to false negatives (because overly strict choice of discovery threshold will mean you take no genes forward to replication). Lastly, another similar point is that because you seek to replicate genes that exceed the significance threshold for EITHER the KS OR SKAT-O test, there is a multiple testing issue. However, again, I believe the high correlation between the two tests means the issue is slight, and this is evidenced by the similarity of the results from each.

So in summary, I think more appropriate to say you are fixing the FWER, rather than the global FDR.**

We have indeed replaced FDR by FWER control of genome-wide error rate in the revised manuscript.

**Further, it would be good to give some justification for 6e-4 discovery threshold (even if if you chose it only for convenience or logistical reasons).**

We now describe the permutation analysis in the main MS for choosing the threshold p-values in the discovery and validation studies (Page 10, line 181).

**Lastly, I would focus on one test, then use the other for a sensitivity analysis (else consider correcting for the fact you are performing multiple tests).**

We used *BF_KS_* as the main test and considered *BF_SKAT_* as secondary sensitivity analysis (Page 9, line 157).

**2 - I would be reassured to see results from a permutation analysis. For example, you could repeat the analyses of ILCC and UKBB 100 times, each time permuting the order of individuals (perhaps using residuals adjusted after allowing for covariates), then record how often one or more genes achieve genome-wide significance - ideally, this should happen at most 5 times (if your control of FWER is effective)**

We thank the reviewer’s suggestion; we have performed the permutation analysis as suggested and found that the permutation results can help determine the threshold of p-values $P_{d}$ and $P_{r}$, which are considered more accurate and robust than using our previous approach, since some slight deviation of the BF distribution from the theoretical null distribution may occur in real data analysis (Page 10, line 180). **Minor comments

3 - I thought the study performed careful QC and it was well explained.**

We thank the reviewer for these positive comments. **4 - I do not fully understand the equation on page 9. Nonetheless, the resulting thresholds (6e-4 and 5e-3) seem reasonable, because their product is close to 3.6e-6.**

We revised the rationale for determining the significance thresholds (see previous replies) (Page 10, line 180).

**5 - I suggest including combined p-values in Tables 3 and 4, eg use Fishers method to combine p-values from discovery and replication analyses.**

We have included combined p-value in Table 3 and 4 using Fisher’s method as suggested (Page 13-14).

**6 - I do not like that your significance threshold for the single RV analysis is FDR q <0.01 (again, because I am not in favour of the using FDR significance thresholds). I would prefer instead using P<0.05/(155+174)=1.5e-5. While it appears no single SNPs achieve this threshold, I do not consider this a major problem, as this is only a secondary analysis intended to give a better understanding of the main results.**

We agree that further validation of the single variant associations is needed. We mainly used FDR as a less conservative approach to highlight the important single variants within the genes discovered compared to the more commonly-used Bonferroni approach.

**7 - I like that you provide a summary of the key maths of the gene-based method in the supplement.**

Thank you.
 **8 - In the abstract / intro, you discuss how common snp gwas have only found 12.3% of LC heritability, giving the impression that rare variants might explain a large share of LC heritability. However, you do not seem to return to this point. Do you think your results suggest they do not (because your two genes have such small effects)? Or maybe the limited sample size (while big for sequence data, an effective sample size of about 4,000 is small for GWAS) means the contribution of rare variants for LC remains an open question?**

We thank the reviewer’s suggestion. It is hard to get a full picture of what could be the contribution of rare variants to lung cancer heritability from our study. Our future direction will be to use UKB and large biobank data as the discovery sample instead of ILCCO and in terms of methodology, to add prior information on single RVs to enhance the power of new genes and RVs discoveries.

**Signed Doug Speed**

**Reviewer #2: This paper reports gene-based association tests using a novel Bayes Factor statistics in ILCCO (a discovery study) and UK Biobank (a replication study). However, a major concern with this method is its inability to control for covariates. Table 2 shows that smoking behavior differs significantly between cases and controls, which is to be expected. However, the Bayes Factor method cannot adjust for this confounding factor. Additionally, other potential confounders, such as top PCs, age, and gender, can compromise the reliability of the analysis results, even though the authors attempted to make these factors comparable between cases and controls. Therefore, it would be advisable for the authors to use established packages like SAIGE-GENE / SAIGE-GENE+ for analysis until the Bayes Factor method can accommodate covariates, as discussed in the Biometrics paper.**

Our Bayes factor approach can easily be extended to account for possible confounding by including covariate information although this extension was not presented in our original Biometrics paper. Here, we described this extension in the S1 Text (Page 3 in Supplemental Information). We have incorporated age, sex, smoking status and top 5 PCs as confounders in the adjusted *BF_KS_* and *BF_SKAT_* for the two top genes CTSL and APOE. We found that the association signal for CTSL did not change much after adjustment for confounders using *BF_KS_* (unadjusted: discovery p-value=4.87E-05, validation p-value=2.75E-03; adjusted: discovery p-value=2.84E-05, validation p-value=3.88E-03) and *BF_SKAT_* (unadjusted: discovery p-value=4.30E-04, validation p-value=1.31E-05; adjusted: discovery p-value=1.32E-03, validation p-value=4.33E-05). While the adjusted association using *BF_SKAT_* on APOE (discovery p-value=2.12E-03, validation p-value=8.24E-03) was not as significant as the unadjusted *BF_SKAT_* (discovery p-value=2.56E-04, validation p-value=4.01E-03). Of note, in this analysis, 9 out of 1923 individuals were removed from ILCCO study due to the missing smoking status and 761 out of 173494 individuals were removed from UK Biobank study due to the missing values of smoking and/or PCs.

**As a minor suggestion, adding p-values in Table 2 instead of only in the text could enhance clarity.**

It is done (Page 11).

**Reviewer #3: This paper utilized Bayes Factor statistic to identify RVs associated with lung cancer using ILCOO as the discovery dataset and UK biobank as the replication dataset. The analysis approach is justifiable and the authors found CTSL and APOE were associated with lung cancer in both studies.**

**My comments on the paper are as follows:
1. Please explain how you identified European ancestry individuals in ILCOO data?**

Principal component analyses (PCA) were performed on ILCOO data along with HapMap controls to establish the ancestry status and to identify possible population outliers. One self-identified white individual (Toronto site, a light blue dot labeled as MtSinai in the following figures) appears to have predominately Asian ancestry, as this sample is clustered with HapMap CHB and JPT control samples in the following figures of top PCs (PC1-PC3). We removed this individual from statistical analyses.

**2. I wonder whether the authors considered the batch effects across different sites in ILCOO data.**

We did not consider batch effect across different sites, as sequencing processes of all DNA samples from four different sites in ILCOO study were performed at the same experiment location, Center for Inherited Disease Research (CIDR). Therefore, the batch effects were not considered in the analyses. We clarified this in the manuscript (Page 7, line 116).

**3. Please specify the specific number of individuals of ILCCO and UK Biobank WES data before and after removing individuals.**

We have added sentences to specify the number of individuals before and after removing individuals in ILCCO study and UK Biobank data (Page 6, line 90; Page 7, line 103; Page 7, line 108 and 112).

**4. I wonder how the authors identified rare variants. What if the MAF of a specific variant is >0.01 in ILCCO and <0.01 in UK Biobank.**

We filtered out variants with MAF>0.01 using “bcftools” software in ILCCO and UK Biobank separately. For the same gene, UK Biobank data usually have more rare variants included, due to its bigger sample size. It is not necessary to have exactly the same set of variants in each study for each gene in gene-based analyses. The association signal is expected to be consistent even though the set of rare variants within the same gene changes across datasets.

**5. What if a specific RV only be found in one dataset? Did you remove it or keep it?**

All variants that passed the QC were kept in our analyses and the number of variants for each gene could vary across the 2 studies, as indicated in the last question. So yes, it is possible that some variants only exist in one dataset.

**6. BF statistics are quite interesting. However, SAIGE-GENE and REGENIE are more popular in gene-based tests. Have you compared your results with the other two methods? Btw, both methods I mentioned can control family relatedness.**

To our knowledge, the main advantage of SAIGE-GENE is that it is applicable to whole-exome sequencing of large Biobank data while accounting for unbalanced case-control ratios and family relatedness. For REGENIE, the efficient computation algorithm can save researchers time and memory in the biobank unbalanced analyses. However, accounting for sample relatedness and improving algorithm efficiency were not the main issues in our study. We have removed related samples from both ILCCO and UK Biobank studies in our QC steps and so that we do not have to worry about sample relatedness in the data analyses. We only used UK Biobank data as validation set for a few identified genes from the discovery set. Therefore, algorithm efficiency was not an issue either.

Finally, the advantages of our BF approach in comparison to competing approaches was discussed in length in the paper J. Xu et al. [11].

**7. Could you please explain why the choice of lambda (0.005) is larger than gamma (6E-4)? The sample size of UK Biobank is much larger than ILCOO. It will be very helpful if the authors can explain why such a choice of lambda and gamma is optimal.**

Inspired by another reviewer’s suggestion, we have adopted a new permutation approach to determine the thresholds in the discovery and validation studies. It is described in Page 10 and Line 172.

**8. The qq plots (Fig 1) look great. Could you please also show qq plots from validation cohorts? When case control ratio is unbalanced, how does the qq plot of BF statistics look like?**

We only used UK Biobank data for the validation analysis and we did not run genome-wide analyses on it. However, we checked the null distribution of BF with/without KS informative prior (600 replicates) under an unbalanced design (N.case=500, N.control=50,000) and the QQ plot shows that the both BF values with and without KS prior follow the theoretical null distribution. In the figure below, the left panel displays the distribution of BF with noninformative prior following Chi-square distribution with 1 degree of freedom; the right panel displays the distribution of BF with KS prior (Fisher’s exact test used for single variant test) approximately follows Chi-square distribution with 3 degrees of freedom, slightly deflated than the theoretical distribution, due to the conservative feature of Fisher’s exact test used for unbalanced design.

**9. Could you explain a little bit about KS and SKAT prior? Which one do the author recommend?**

*We have included this text in the S1 Text* (Page 3):

*“BF_KS_* and *BF_SKAT_* statistics might prove useful for both gene-based and single RV-based association studies. Indeed, the BF with noninformative prior compares the overall RV count between cases and controls, whereas the KS component in *BF_KS_* is based on allelic distribution differences between cases and controls at individual sites within the region, and the SKAT component in *BF_SKAT_* is based on the contribution of each RV site to the phenotype variability.”

**10. I am curious how the authors derived p-values from Bayes factor? P-values are usually not available in Bayesian methods.**

P-values can be derived even from Bayesian statistics as long as a null distribution is available. We have derived the null distribution of BF with KS test as informative prior in the paper Xu, J et al. [11]. We clarified the null distribution of BF at page 2 in the S1 Text.

**11. Firth biased-corrected logistic regression may not perform well when MAC is too small. It’s hard to believe these p-values (except rs112682750) in Table 5 given # carriers. I will suggest the authors perform resampling methods or efficient resampling when # carriers is less than 20.**

Resampling methods would not solve the problem here because fitting a standard logistic model in each permuted sample would lead to very large bias, the overall estimate then being an average of all biased estimators. We agree that the biased-corrected logistic regression might be improved in this context but in the meantime should provide more reliable estimates than alternative strategies.

**12. I saw you mentioned data source in Data Availability section. Please add it in the manuscript as well.**

It seems the Data Availability section is published automatically with the paper, as per instructions to authors.

**Have all data underlying the figures and results presented in the manuscript been provided?
Large-scale datasets should be made available via a public repository as described in the *PLOS Genetics*** [**data availability policy**](http://journals.plos.org/plosgenetics/s/data-availability)**, and numerical data that underlies graphs or summary statistics should be provided in spreadsheet form as supporting information.**

**Reviewer #1: Yes**

**Reviewer #2: None**

**Reviewer #3: Yes**

**PLOS authors have the option to publish the peer review history of their article (**[**what does this mean?**](https://journals.plos.org/plosgenetics/s/editorial-and-peer-review-process#loc-peer-review-history)**). If published, this will include your full peer review and any attached files.


If you choose “no”, your identity will remain anonymous but your review may still be made public.**
